# The Impact of Vitamin D Deficiency on Infants’ Health

**DOI:** 10.3390/nu15204379

**Published:** 2023-10-16

**Authors:** Andreea Bianca Stoica, Claudiu Mărginean

**Affiliations:** 1 Doctoral School of Medicine, George Emil Palade University of Medicine, Pharmacy, Science and Technology of Târgu Mureș, Gheorghe Marinescu Street No. 38, 540136 Târgu Mureș, Romania; 2 Department of Obstetrics and Gynecology 2, George Emil Palade University of Medicine, Pharmacy, Science and Technology of Târgu Mureș, Gheorghe Marinescu Street No. 38, 540136 Târgu Mureș, Romania; marginean.claudiu@gmail.com

**Keywords:** vitamin D, vitamin D deficiency, infants, pregnancy, health status

## Abstract

Vitamin D is an essential nutrient that plays a vital role in bone health and musculoskeletal development. The aim of this narrative review is to present up-to-date information about the impact of vitamin D deficiency (VDD) on the health status of infants in their first year of life. Vitamin D is indispensable for skeletal growth and bone health, and emerging research suggests that it may also have significant roles in maternal and fetal health. VDD affects a large proportion of infants according to current guidelines. However, its prevalence varies depending on geographic location, skin pigmentation, and the time of year. Based on current guidelines for normal vitamin D levels and recommended daily intake, studies suggest that VDD is a global health issue with potentially significant implications for those at risk, especially infants. Our understanding of the role of vitamin D has improved significantly in the last few decades. Systematic reviews and meta-analyses investigating the effect of vitamin D on preterm birth, low birth weight, anthropometric parameters, and health outcomes such as infectious diseases in infants, have found conflicting or inconsistent results. It is important to encourage further research to fill in these knowledge gaps and develop national or global strategies that ease the burden of VDD, especially in groups at risk.

## 1. Introduction

Vitamin D is an essential nutrient that plays a vital role in bone health and musculoskeletal development. In recent years, a large number of studies have highlighted the involvement of vitamin D in the regulation of blood pressure, glycemia, and immune response, and its significant role during pregnancy [1].

However, the results of these studies are often contradicting, and there are uncertainties regarding the association between vitamin D deficiency (VDD) and certain health outcomes. Furthermore, there is a lack of consensus among professional organizations regarding the optimal level of vitamin D in the body and the ideal doses for supplementation. Data suggests that VDD is a significant health issue in many parts of the world, and the risk factors for VDD are prevalent even in areas where sun exposure is adequate [2]. Infants are particularly at risk of VDD given their dependence on the vitamin D status of their mother, the vitamin D content of breastmilk, and the fact that their exposure to the sun may be limited due to safety reasons or cultural norms. The aim of this narrative review is to present up-to-date information about the impact of vitamin D deficiency on the health status of infants in their first year of life.

## 2. Physiology and Roles in the Body

Vitamin D is a fat-soluble vitamin that is mainly synthesized in the skin from 7-dehydrocholesterol upon exposure to ultraviolet B (UVB) (290–320 nm) radiation from sunlight. Although unconstrained UVB exposure would provide adequate serum levels, vitamin D3 synthesis in the skin is diminished or absent at latitudes above 35°, particularly during the winter, and due to individual factors, such as darker skin pigmentation or limited UVB exposure [2]. Vitamin D can also be obtained from the diet, in the form of vitamin D2 (ergocalciferol) from plant sources or vitamin D3 (cholecalciferol) from animal sources. However, vitamin D is naturally present in only a few foods such as fish liver oils, fatty fish (salmon, swordfish, tuna, sardines), liver, mushrooms, and egg yolks [3]. Vitamin D3 is the main form of vitamin D, also found in most commercial supplements. In the liver, the enzyme 25-hydroxylase converts vitamin D to 25-hydroxyvitamin D [25(OH)D], the main circulating form of vitamin D, which is then transformed in the kidneys by 1α-hydroxylase to the active metabolite 1,25-dihydroxyvitamin D [1,25(OH)2D].

The most important function of vitamin D is the insurance of calcium and phosphate homeostasis, which is essential for bone growth and bone mineral metabolism. Serum calcium levels are regulated through a unique relationship between vitamin D and parathyroid hormone (PTH). Low calcium levels stimulate the release of PTH, which increases 1,25(OH)2D production and calcium reabsorption in the kidney and mobilizes calcium and phosphorus from the bones [4]. Vitamin D inhibits PTH and improves calcium status by increasing the intestinal absorption of calcium and phosphorus [5]. Through its actions, vitamin D increases bone density [6] and may have an important role in the prevention of osteoporosis [7]. In addition to the development, function, and maintenance of healthy bones, vitamin D is also thought to be involved in the regulation of neuromuscular function, improving muscle strength [8] and balance [9].

The extraskeletal roles of vitamin D are suggested by the fact that vitamin D receptors are present in multiple organs and systems throughout the body [10], and 1α-hydroxylase is also present in various organs including the placenta, bone, and parathyroid gland [11]. Immune cells not only feature vitamin D receptors [12] but are also able to convert vitamin D3 to its active metabolite [13]. Studies suggest that vitamin D potentiates the innate immune system and regulates the adaptive immune system [14]. This may explain its potential involvement in the prevention of respiratory infections [15], urinary tract infections [16], and tuberculosis [17], but also in certain diseases with an autoimmune background such as diabetes [18] or multiple sclerosis [19].

In the nervous system, vitamin D receptors are present in the hippocampus, hypothalamus, thalamus, subcortical grey nuclei, and substantia nigra, and vitamin D is believed to regulate neural differentiation and maturation [20]. It is also believed that VDD may be associated with neurological diseases such as multiple sclerosis [21], ischemic stroke [22], and Parkinson’s disease [23]. In the pancreas, vitamin D binds to its receptors on beta cells and helps maintain normal blood glucose levels by regulating the release of insulin [24,25]. VDD may also be associated with an increased risk of cardiovascular diseases, and large population studies suggest a possible link between VDD and hypertension [26], myocardial infarction [27], stroke [28], and heart failure [29]. However, most of this data comes from observational studies, and causality will have to be proven by large-scale, long-term randomized controlled trials.

During pregnancy, the calcium metabolism of the mother suffers significant changes in order to enable the accretion of calcium in the fetal skeleton while keeping maternal serum calcium concentrations stable [30,31]. These changes include the increase in calcium absorption in the intestines [32,33] and the mobilization of calcium from the maternal skeleton [34]. Vitamin D is thought to have an important contribution to this process, as 1,25(OH)2D levels increase in the second and third trimesters [32]. On the other hand, the effect of pregnancy on 25(OH)D is less well understood; some studies show a reduction through pregnancy [35], while others have not been able to demonstrate significant changes compared to pre-pregnancy levels [33]. Maternal 25(OH)D levels are important, as the 25(OH)D status of the fetus depends entirely on that of the mother [30].

## 3. Vitamin D Deficiency

### 3.1. Definition

The best indicator of vitamin D status is 25(OH)D serum level due to its long half-life, relative stability, and responsiveness to endogenous vitamin D production or exogenous intake [4]. Serum 25(OH)D can be measured by liquid chromatography–mass spectrometry or enzyme-linked immunosorbent assay, although there can be significant differences between laboratories and the technique used [4]. Although 25(OH)D is a reliable biomarker, it functions more as an indicator of recent vitamin D exposure rather than of functional outcomes or health effects, which has to be considered when evaluating the potential link between vitamin D status and certain diseases [4]. Furthermore, according to Roth et al. (2018), many illnesses may decrease 25(OH)D levels and observed associations between diseases and 25(OH)D levels may be owed to reverse causation [4].

Based on the available evidence, different professional organizations have used different 25(OH)D values as thresholds for vitamin D deficiency, insufficiency, and sufficiency. The Institute of Medicine (IOM) set the cut-off value for vitamin D deficiency at a 25(OH)D level <30 nmol/L, for inadequacy at 30–50 nmol/L, and for sufficiency at >50 nmol/L [36]. The European Food Safety Authority (EFSA) set the cut-off value for deficiency at <50 nmol/L and for sufficiency at >50 nmol/L [37], and the Endocrine Society set the cut-off value for deficiency at <50 nmol/L, for insufficiency at 50–70 nmol/L, and for sufficiency at >70 nmol/L [38].

It has to be noted though that as 25(OH)D alone does not accurately predict health outcomes, most experts agree that a single threshold for deficiency is unlikely to be valid in all situations, and the interpretation of 25(OH)D values should always take into account age, ethnicity, genetics, calcium intake, inflammation, and other factors that may influence vitamin D status [4].

### 3.2. Prevalence

The global prevalence of VDD may be difficult the estimate because representative 25(OH)D data are missing for many countries, especially for low- and middle-income countries (LMICs) [39], and the data that does exist may present an incomplete picture that does not take into account variations related to dietary intake and UVB exposure [4]. That said, the prevalence of low vitamin D status varies significantly by geographic location. In a meta-analysis of 308 studies with almost 8 million participants from 81 countries, Cui et al. (2016) found serum 25(OH)D levels < 30 nmol/L in 15.7% of the enrolled population and <50 nmol/L in 47.9% of individuals [40]. In the US, Schleicher et al. (2016) found 25(OH)D levels < 30 nmol/L in 5.9% and <50 nmol/L in 24% of the population [41]. In Europe, Cashman et al. (2022) found 25(OH)D levels < 30 nmol/L in 13% and <50 nmol/L in 40% of the population [42]. Data suggest that the prevalence of VDD may be the highest in Asia, the Middle East, and Africa. Recent data from 119 studies found serum 25(OH)D levels < 30 nmol/L in 18.5% and <50 nmol/L in 34.2% of individuals in Africa [43]. As far as the Middle East and Asia are concerned, Palacios et al. (2014) reported 25(OH)D values < 30 nmol/L in 51% of infants in Turkey, 86% of infants in Iran, and 61% of infants in India and Pakistan [44].

### 3.3. Groups at Risk

VDD affects individuals in all age groups worldwide. Vitamin D status is influenced by a multitude of factors that include diet and supplement use, age, geographic latitude, cultural and lifestyle factors, skin pigmentation, and individual differences in vitamin D metabolism [4,42,44].

One of the most important determinants of vitamin D status is the amount of vitamin D produced in the skin as a response to UVB exposure. Individuals who spend less time in the sun (e.g., have limited mobility, such as the elderly, or spend most of their day indoors) [45,46], have darker skin [47], or use sunscreen or protective clothing [48], are more exposed to the risk of VDD.

Socioeconomic factors, such as marital status, education, and income, can also influence vitamin D status by affecting sunlight exposure, dietary choices, or access to healthcare. In a study of 1151 healthy women aged between 18 and 40 years from rural China, a lower socioeconomic status, defined as the women’s education level and occupations, their husband’s education level and occupation, as well as household income and expenditure, was associated with a higher risk of VDD or insufficiency [49]. Similarly, a study investigating the prevalence of VDD and associated risk of all-cause and cause-specific mortality among middle-aged and older adults in the United States found that social determinants such as marital status, educational attainment, and family income to poverty ratio may have a role in serum vitamin D levels, participants with a lower socioeconomic status being more likely to have VDD [50].

Infants are particularly vulnerable to VDD in their first year of life, as their vitamin D status depends on the status of their mother, the vitamin D content of breast milk, and whether or not they receive vitamin D supplements [51]. They are also more protected from the sun due to safety concerns or cultural norms [38]. Other risk factors for VDD in infancy include female gender, having a multiparous mother, and being exclusively breastfed. Furthermore, these risk factors seem to exhibit a cumulative effect [52]. However, the most important factor that affects the vitamin D status of the infant is the vitamin D status of the mother.

### 3.4. Intake Recommendations

Current recommendations regarding vitamin D intake vary depending on age and physiological status. Different professional organizations have established different vitamin D intake levels for their target populations; however, recommended intakes are generally between 5 and 20 μg/day, assuming minimal sunlight exposure. The World Health Organization (WHO) recommends 5 μg/day for infants, children, and adults < 50 years, 10 μg/day for 51–65 years, and 15 μg/day > 65 years in order to maintain 25(OH)D levels at 27 nmol/L or above [53]. In the US, the IOM recommends 10 μg/day for infants, 15 μg/day for children and adults, and 20 μg/day for the elderly in order to maintain 25(OH)D levels at 50 nmol/L or above [36]. In Europe, the EFSA recommends 10 μg/day for infants and 15 μg/day for all other age groups in order to maintain 25(OH)D levels at 50 nmol/L or above [37]. The majority of guidelines do not recommend higher vitamin D intake during pregnancy.

Recommendations regarding vitamin D intake must also consider the risk of toxicity at very high intake levels. Vitamin D toxicity results in hypercalcemia and/or hypercalciuria, which may be accompanied by impaired renal function and increased intestinal absorption of calcium and bone resorption [54]. Therefore, the IOM [36] and the EFSA [37] recommend an upper intake level of 4000 IU/day (100 μg) in adults and children aged above 9 and 11 years, respectively. In 2018, the Panel on Dietetic Products, Nutrition and Allergies of the EFSA updated its recommendation regarding the tolerable upper intake level of vitamin D for infants, increasing it to 35 µg/day for infants aged 6–12 months, while the tolerable upper intake level for infants < 6 months remained at 25 µg/day [55].

## 4. Vitamin D Deficiency in Infants

### 4.1. Prevalence

In a 2020 study investigating the vitamin D nutritional status of infants in Northern Taiwan, Chen et al. (2020) found that 41% of infants had VDI and 44% had VDD based on the guidelines of the Endocrine Society [56]. The study also found that exclusively breastfed infants had a higher prevalence of VDI (86.1%) compared to mixed-fed (51.9%) and formula-fed (38.5%) infants, and the prevalence of VDD was 55.4% in infants aged under 6 months and 61.6% in infants aged over 6 months. In a systematic review of 30 studies examining VDD in Southeast Asian children, Oktaria et al. (2022) found that the prevalence of VDD ranged from 0.9% to 96.4% based on a cut-off value of <50 nmol/L, with more than 50% of newborns having VDD [57]. In another study published in 2020, the same authors found that VDD had a prevalence of 90% in the cord blood samples of a group of 344 Indonesian newborns [58]. In a US study on 380 healthy infants and toddlers aged between 8 and 24 months, Gordon et al. (2008) found a prevalence of VDD of 12.1%, whereas 40% of infants had 25(OH)D levels below the 30 ng/mL threshold [59]. In an Indian study published in 2020, Chacham et al. (2020) found that 74% of the 200 included infants had VDD [60]. In another study carried out in India, Kumar et al. (2020), found that from 825 healthy newborns, 92.1% had VDD at birth [61]. In the Netherlands, Hoevenaar-Blom et al. (2019) found that in the winter, 32% of infants had VDD at a cut-off value of 50 nmol/L [62]. In a study conducted in China, the authors found that VDD had a prevalence of 10.8% [63]. In a study conducted on 98 infants in Kenya, the prevalence of VDD was 11.2% at a cut-off value of 12 ng/mL [64]. In every study that compared infants who were breastfed only and infants who also received formula, the prevalence of VDD was higher in exclusively breastfed infants.

### 4.2. The Impact of VDD

The most common effects of VDD are related to bone and musculoskeletal health and are represented by rickets in children and osteomalacia in adults. Nutritional rickets is a disorder characterized by defective chondrocyte differentiation and mineralization of the growth plate caused by low vitamin D status and inadequate dietary calcium intake in children [4]. The manifestations of rickets include impaired linear growth, chest wall deformity, fractures, bone pain, leg deformities, and developmental delay of gross motor skills [65]. Similarly, in adults, osteomalacia is also the result of defective mineralization due to inadequate calcium or phosphorus availability or excessive calcium resorption due to severe VDD, manifested as pain, fractures, and muscle weakness [4]. Due to the low calcium levels it causes, VDD can also lead to seizures, growth failure, irritability, lethargy, muscle weakness, and frequent respiratory infections in infants [66,67].

In recent years, new research suggests that vitamin D may also influence other health outcomes in pregnant women, infants, and children due to its potential involvement in the regulation of bone density and muscle strength, blood pressure, glycemia, immune response, and mood [68]. As a result, VDD has been linked with pre-eclampsia, gestational diabetes, preterm birth, and postpartum depression in pregnant women, and with low birth weight, lower bone mass, respiratory tract infections (RTIs), and asthma exacerbations in infants and children [69].

Studies have reported an increase in lower RTIs, primarily pneumonia and bronchiolitis, in newborns with 25(OH)D < 50 nmol/L compared to 25(OH)D > 75 nmol/L [70], as well as a significant association between low 25(OH)D status and the risk to develop a lower RTI [71]. Moreover, significant correlations were found between 25(OH)D status and disease severity [72], need for intensive care unit admission [73], and longer length-of-stay in infants with bronchiolitis [74]. A large meta-analysis found that vitamin D supplementation was effective in reducing the frequency of upper RTIs in patients of all ages, particularly in those who started supplementation at a 25(OH)D level < 25 nmol/L [75]. Furthermore, a meta-analysis of vitamin D supplementation trials found a significant reduction in the frequency of asthma exacerbations requiring corticosteroid treatment [76]. The effect of vitamin D in these respiratory outcomes is probably mediated, at least in part, by its immune-modulatory effects and the induction of innate immune responses to respiratory viruses [4].

### 4.3. Intervention Strategies

Given that vitamin D can be found naturally in a very limited number of foods, the most widely used strategies to improve vitamin D status and reduce the burden of VDD are fortification and supplementation. Fortification involves adding vitamin D to widely consumed, culturally appropriate foods to increase vitamin D intake. The most effective method is systematic or government-mandated fortification, which ensures that 100% of the food product is fortified with appropriate formulations of vitamin D [77] and also provides regulatory monitoring and enforcement [78]. By contrast, voluntary fortification is less effective as it depends mainly on food producers and may lead to variations in vitamin D content among different products [79]. The most commonly fortified foods are milk and dairy products [80], but oils [81], flours [82], and fruit juices [83] have also been used as vehicles for fortification.

In order to develop policies regarding vitamin D fortification, reliable information is needed on the most effective and safe dosage, the optimal dosing regimen, the timing of initiation, and the effect of vitamin D when combined with other vitamins and minerals [5]. However, as several studies have found, the level of evidence is insufficient to guide policy recommendations [4]. As a result, very few countries (US, Canada, India, and Finland) have introduced systematic fortification [84], and there are data that suggest that VDD may persist despite fortification [44].

Although there is considerable debate regarding the thresholds for deficiency and sufficiency, given the potential effect of VDD on a wide range of health outcomes and the relatively low cost of vitamin D supplementation [85], several guidelines recommend supplementation during pregnancy in daily doses between 400 and 600 IU [37,38,86], However, according to more recent studies that examined the possible link between vitamin D status and infant health outcomes, the presence of this link and the benefits of supplementation do not seem to be entirely supported by evidence.

In a systematic review and meta-analysis of 43 trials with a total of 8,406 participants, Roth et al. (2017) found that vitamin D supplementation during pregnancy increased maternal and cord blood 25(OH)D concentrations, mean birth weight, and infant length at one year of age, and at the same time reduced the risk of small for gestational age and the risk of wheezing in the offspring [87]. These results suggested that an improved prenatal vitamin D status could reduce the prevalence of low birth weight and stunting. However, the authors concluded that most of the trials were small and of low quality, and they found the evidence to be insufficient to guide clinical or policy recommendations [87].

In a randomized, double-blind, placebo-controlled trial conducted on 1164 infants from Bangladesh, Roth et al. (2018) investigated the effect of maternal vitamin D supplementation from 17–24 weeks gestation until birth or 6 months postpartum [88]. Participants were randomly allocated to receive vitamin D in doses ranging from 0 IU/week to 28,000 IU/week until birth or 28,000 IU/week in pregnancy and in the first 6 months postpartum. The study found that prenatal or postpartum maternal vitamin D supplementation had no influence on infant length or other anthropometric outcomes, such as head circumference, upper arm length, mid-upper arm circumference, rump-to-knee length, and weight, by one year of age [88]. Similarly, in the study of Chen et al. (2020), vitamin D status did not influence anthropometric parameters, which were similar in all groups [56]. These results are consistent with those reported by high-quality randomized controlled trials [89,90,91,92]. By contrast, smaller studies suggested that vitamin D status in the second half of pregnancy is a determinant of newborn size [87,93]. However, in these studies, postnatal growth was a post hoc outcome, and the differences observed between groups may have been due to chance.

In a 2019 Cochrane Review, after analyzing data from 22 trials involving 3725 pregnant women Palacios et al. (2019) found that vitamin D supplementation during pregnancy probably reduces the risk of pre-eclampsia, gestational diabetes, and low birthweight, and it may make little or no difference in the risk of having a preterm birth [5]. The authors noted that their grading of recommendation, assessment, development, and evaluation (GRADE) assessments of the included studies ranged from moderate to very low because of limitations in study design, imprecision, and indirectness [5].

In a prospective, randomized, double-blind, placebo-controlled trial published in 2022, oral supplementation with 400 IU/day in 72 exclusively breastfed infants had a significant impact on weight and head circumference, as well as 25(OH)D, phosphorus, and parathyroid hormone levels, measured after 4 months. However, the body length and bone mineral density of infants receiving vitamin D did not differ significantly from those who received a placebo [94]. Similar results were obtained in a Cochrane Review investigating the effect of vitamin D supplementation on linear growth and other health outcomes among children under 5 years of age [94]. The authors reviewed 75 studies with more than 12,000 participants from India, the USA, and Canada, who received daily oral vitamin D doses ranging from 200 to 2000 IU. The study concluded that oral vitamin D supplementation may yield little to no difference in linear growth, stunting, hypercalciuria, or hypercalcemia, but may result in a slight increase in length/height-for-age z-score [94]. However, many of the included studies had small sample sizes, were heterogeneous in terms of population and intervention parameters, and exhibited a high risk of bias.

A systematic review commissioned to support the expert group charged with updating the vitamin D intake recommendations of the Food and Agriculture Organisation of the United Nations (FAO) and WHO for children aged 0–4 years examined 146 studies regarding the effects of different vitamin D intake levels on a variety of health outcomes including infectious disease, growth, neurodevelopment, rickets, and bone mineral density [95]. The review also aimed to investigate the association between serum 25(OH)D levels and health outcomes, and the effect of vitamin D intake on serum 25(OH)D concentrations. The study found that the strength of evidence was low or very low for most outcomes, and it was moderate only for the effect of daily vitamin D supplementation on raising serum 25(OH)D concentrations [95].

Although there is conflicting evidence regarding the benefits of vitamin D supplementation, some studies suggest that it may prevent RTIs during the first year of life. In a 2020 study, doses of 400–600 IU/day from birth to the outcome endpoint were able to delay the occurrence of the first RTI [96]. In infants without supplementation, the median time to the first RTI was 60 days after birth, while in infants who benefited from vitamin D supplementation, the median time to the first RTI episode was longer than 6 months (*p* < 0.001). The authors also observed an inverse relationship between the frequency of supplementation and the risk of RTI and RTI-related hospitalization [96].

### 4.4. Maternal vs. Infant Supplementation

Recent studies examined whether the optimal vitamin D status of infants can be achieved by maternal supplementation alone or maternal plus infant supplementation is needed. Vitamin D is secreted into breast milk, but the concentration depends on the lactating woman’s regular vitamin D intake [88]. According to Hollis et al. (2015), maternal supplementation may require daily doses of 4000 IU/day or higher to boost vitamin D content in breast milk enough to ensure that the equivalent of 200–400 IU/day is transferred to the exclusively breastfeeding infant [97].

In a study carried out on 95 exclusively breastfeeding mothers and their infants in Qatar, Dawodu et al. (2019) compared the effect of maternal vitamin D supplementation of 6000 IU/day alone with maternal supplementation of 600 IU/day plus infant supplementation of 400 IU/day over a period of 6 months. Women receiving 6000 IU/day achieved adequate serum 25(OH)D levels in a proportion of 96% compared to 52% of the women receiving 600 IU/day (*p* < 0.0001). However, infants supplemented with 400 IU vitamin D had slightly higher serum 25(OH)D levels than those who received their vitamin D through breastfeeding only [98]. In a study published in 2018, Aghajafari et al. (2018) also concluded that both mother and infant require vitamin D supplementation to ensure that the infant’s vitamin D status is adequate based on current guidelines [99]. However, studies suggest that in both high- and low-income countries, maternal supplementation may be preferred to infant supplementation [88].

### 4.5. Cholecalciferol vs. Calcifediol

Although vitamin D supplementation is usually performed using vitamin D3 (cholecalciferol), studies have also investigated the benefits of using 25(OH)D (calcifediol) for supplementation, given its more efficient intestinal absorption and greater bioavailability. A meta-analysis of nine RCTs found oral calcifediol to be about three times more potent than oral cholecalciferol, resulting in a more rapid increase in 25(OH)D serum levels and the need for lower doses to achieve the same effect as cholecalciferol [100]. However, calcifediol is currently not used for vitamin D supplementation, in infants or adults, and the authors concluded that more research is needed to confirm its potential advantages over cholecalciferol [101].

### 4.6. Current Situation

The results of recent studies investigating the effects of maternal VDD on infant outcomes have underlined the uncertainties about vitamin D requirements in pregnancy and lactation, and there is still a lack of consensus regarding the association between vitamin D status and infant health outcomes. Although most guidelines recommend vitamin D supplementation during pregnancy and the postpartum period, the evidence presented in these studies does not support routine vitamin D supplementation even in communities with endemic vitamin D deficiency and infant growth restriction [88]. These recommendations are consistent with those of the WHO. In its 2016 guideline, the WHO did not recommend the routine supplementation of vitamin D during pregnancy due to lack of evidence, and only in cases of VDD [53]. In response to new evidence regarding these interventions, in 2020 the WHO updated its antenatal nutrition guideline. Still, the initial recommendations have not been changed, and the WHO continues to recommend vitamin D supplementation only for pregnant women with suspected VDD, at a daily dose of 200 IU, including populations where sun exposure is limited [102]. A summary of the clinical studies that assessed the impact of vitamin D deficiency and its subsequent supplementation in infants has been provided in Table 1.

## 5. What Is Next?

In 2017, a working group convened by the Sackler Institute for Nutrition Science at the New York Academy of Sciences and the Bill & Melinda Gates Foundation to assess the global prevalence and disease burden of vitamin D deficiency outlined numerous knowledge gaps related to the role of vitamin D in specific health outcomes. These gaps include: the extent of vitamin D deficiency, especially in LMICs; vitamin D and calcium intake, especially in LMICs; the effects of low vitamin D status and vitamin D supplementation on child morbidity and mortality; the dose-dependent effects of vitamin D supplementation on maternal and infant outcomes; the relationship between vitamin D and other nutrients related to pregnancy outcomes and the risk of rickets; the dose and regimen of maternal supplementation in order to optimize 25(OH)D levels in breastfed infants; the potential benefits of vitamin D in the prevention of respiratory infections and the treatment of asthma and tuberculosis [4]. Addressing these knowledge gaps must start with the basics, i.e., reaching a common ground regarding the definition of vitamin D deficiency, and gathering real data about its national, regional, and global prevalence. High-quality randomized controlled trials are needed to determine if there really is a link between vitamin D deficiency and certain health outcomes. If there is, then supplementation guidelines should be harmonized and a roadmap should be developed to reduce the burden of vitamin D deficiency, especially in LMICs. Long-term studies are needed to identify the genetic factors that influence vitamin D status and response to supplementation, develop evidence-based guidelines regarding supplementation, assess the risk-benefit ratio of high-dose vitamin D supplementation, evaluate the effects of vitamin D supplementation on bone health and immune function, establish the causal relationship between vitamin D and certain chronic diseases. We should identify the clinical and genetic markers that can guide individualized supplementation and develop targeted interventions to address socioeconomic and racial disparities in vitamin D status. In addition, future studies should answer the question of whether vitamin D deficiency is a public health problem in LMICs or merely a condition that affects certain groups with high risk. Scientists also need to find ways to increase awareness among the general public on the impact of vitamin D on health outcomes, improve adherence to infant and childhood vitamin D supplementation, and address the current limitations of supplementation such as the issue of individual response to therapy.

Special attention should be given to healthcare providers, who have a substantial role in decreasing the burden of VDD by identifying individuals at risk, educating patients regarding the importance of vitamin D, sunlight exposure, a balanced diet, and vitamin D supplementation, increasing patient adherence, as well as providing monitoring and follow-up. Furthermore, fortification and supplementation efforts must be accompanied by public health campaigns to increase awareness of the importance of vitamin D.

## 6. Conclusions

Vitamin D is indispensable for skeletal growth and bone health, and emerging research suggests that it may also have significant roles in maternal and fetal health. Based on current guidelines for normal vitamin D levels and recommended daily intake, studies suggest that VDD is a global health issue with potentially significant implications for those at risk, especially infants. Although our understanding of the role of vitamin D has improved significantly in the last few decades, there are still important knowledge gaps regarding the true impact of VDD. Almost every aspect of VDD, from its real prevalence to the optimal supplementation regimen, needs further research and reliable evidence. These issues are further complicated by the fact that the concurrent deficiency of other nutrients can confound the assessment of health outcomes related to VDD, especially in LMICs, where multiple nutrient deficiencies are often prevalent. Although it seems logical to increase vitamin D intake in those who are deficient or are at risk of becoming deficient, most systematic reviews and meta-analyses investigating the effect of vitamin D on a series of maternal and infant outcomes found that vitamin D supplementation during pregnancy does not have a significant effect on linear growth and stunting, or anthropometric parameters such as head circumference, upper arm length, mid-upper arm circumference, rump-to-knee length, and weight. These studies also concluded that vitamin D supplementation during pregnancy probably reduces the risk of pre-eclampsia, gestational diabetes, and low birth weight, but it may make little or no difference in the risk of having a preterm birth. However, most meta-analyses reported that many of the included studies had small sample sizes and were poorly designed, therefore the evidence was insufficient to guide clinical or policy recommendations. As a consequence, there is uncertainty regarding the impact of VDD on infants, and there is a lack of consensus regarding the optimum dosage for vitamin D supplementation. Most guidelines recommend vitamin D supplementation during pregnancy with daily doses between 400 and 600 IU, but currently, these recommendations seem to be based on the lack of adverse effects rather than clear evidence regarding the benefits. It is important to encourage further research to fill in these knowledge gaps and develop national or global strategies that ease the burden of VDD, especially in groups at risk.

## Figures and Tables

**Table 1:** Characteristics of clinical studies which assessed vitamin D deficiency in infants.

Reference (Author, Year)	Study Design	Population and Study Group Assignment	Duration	Main Outcome	Number of Patients	Vitamin D Used Dose
Tung et al., 2021 [52]	Cross-sectional	Infants aged 2 to 6 months	2 years	The risk of vitamin D insufficiency could be higher among breastfed infants who experience more risk factors.	208	400 IU/d
Chen et al., 2000 [56]	Cross-sectional	Infants in the first year of life	3 years	Dietary vitamin D intake and birth season were major predictors of vitamin D deficiency.	481	400 IU/d
Oktaria et al., 2020 [58]	Cohort	Newborn and infants aged 6 months	2 years	Risk factors for vitamin D deficiency at six months of age were insufficient exposure of infant’s skin area to direct sunlight, severe vitamin D deficiency at birth, and EBF until six months of age.	350	400 IU/d
Gordon et al., 2008 [59]	Cross-sectional	Infants and toddlers aged 8 to 24 months	2 years	Breastfeeding without vitamin D supplementation among infants and lower milk intake among toddlers were significant predictors of vitamin D deficiency.	380	200 IU/d
Hoevenaar-Blom et al., 2019 [62]	Cross-sectional	Children aged 6 to 48 months	1 year	Determinants of vitamin D deficiency in infants and toddlers were winter, age over two years, lack of formula feeding, absent or low vitamin D supplementation and overweight.	150	10 ug/d
Mansur et al., 2022 [69]	Systematic review	Pregnant women and newborns	1 year	Ensuring appropriate supplementation in children with vitamin D deficiency reduces the risk of respiratory infections, possibly of autoimmune diseases and autism.	2487	400 IU/d; 1000 IU/d; 2000 IU/d; 2800 IU/d; 4000 IU/d
Hong et al., 2020 [96]	Cohort	Newborns and infants in the first 6 months	2 years	Infants benefiting from vitamin D supplementation had longer periods of time free from respiratory tract infections when compared with those infants who did not receive any supplements	2244	400–600 IU/d
Hollis et al., 2015 [97]	Cross-sectional	Pregnant women and newborns	2 years	Vitamin D supplementation in the adequate dose of breastfeeding mothers satisfies the nursing infant’s requirement.	334	400 IU/d
Aghajafari et al., 2018 [99]	Cohort	Pregnant women and newborns	3 years	To ensure optimal vitamin D status, not only do infants require a supplement, but women also need to take vitamin D supplements during breastfeeding.	500	400 IU/d

## Data Availability

Not applicable.
